# Integrating transcriptomic analysis and experimental validation unravels autophagy- and lactylation-related gene signatures in acute myocardial infarction

**DOI:** 10.3389/fcvm.2026.1814910

**Published:** 2026-10-07

**Authors:** Jun Peng, Li Chen, Lizhen Liu, Xiangdong Xu, Huiwen Song

**Affiliations:** 1 Department of Cardiology, The First People's Hospital of Xiaoshan District, Xiaoshan Affiliated Hospital of Wenzhou Medical University, Hangzhou, China; 2 Department of Cardiology, Jiading District Central Hospital Afﬁliated Shanghai University of Medicine & Health Sciences, Shanghai, China

**Keywords:** acute myocardial infarction, autophagy, biomarkers, lactylation, machine learning

## Abstract

**Background:**

Acute myocardial infarction (AMI) is a leading global health burden. While autophagy and lactylation are crucial in cardiovascular pathophysiology, their coordinated roles in AMI remain unclear. This study investigates the transcriptional signatures associated with both processes to identify diagnostic biomarkers.

**Methods:**

Transcriptomic datasets (GSE66360, GSE48060), autophagy-related genes (ARGs), and lactylation-related genes (LRGs) were analyzed. Differentially expressed ARGs and LRGs (DE-ARGs, DE-LRGs). Weighted gene co-expression network analysis (WGCNA) pinpointed key module genes correlated with their activity scores. Biomarker selection employed protein-protein interaction networks, least absolute shrinkage and selection operator (LASSO) regression, artificial neural network (ANN) model, and validation in clinical samples.

**Results:**

WGCNA identified 1,442 key module genes. ATM and STAT1 were validated as robust diagnostic biomarkers. Models built with these genes showed high diagnostic accuracy in independent cohorts. Functional enrichment implicated the neuroactive ligand-receptor interaction pathway. Immune infiltration analysis revealed a statistically significant negative correlation between ATM/STAT1 expression and eosinophil infiltration levels (cor=−0.42, *p* < 0.05), suggesting a potential association. Rat models showed a trend toward downregulation of both biomarkers in AMI, although these findings require validation in larger cohorts due to the limited sample size (*n* = 4 per group).

**Conclusions:**

This integrative study identifies ATM and STAT1 as promising diagnostic biomarkers for AMI, with their expression signatures robustly correlated with both autophagy- and lactylation-associated gene activity scores. These findings provide a foundation for further investigation into the potential roles of these genes in linking metabolic reprogramming and inflammatory responses in AMI.

## 1 Introduction

Acute myocardial infarction (AMI) is a critical condition triggered by the rupture of atherosclerotic plaques in the coronary arteries, leading to thrombosis and subsequent acute, persistent myocardial ischemia and hypoxia, which ultimately results in myocardial necrosis (1–3). As one of the leading causes of death worldwide, it is characterized by sudden onset, rapid progression, and frequent complications such as malignant arrhythmias, heart failure, or cardiogenic shock, posing a serious threat to life (4–7). Currently, the diagnosis of AMI primarily relies on characteristic electrocardiographic changes and dynamic monitoring of serum myocardial injury markers, especially cardiac troponin. Treatment focuses on achieving coronary reperfusion as quickly as possible, such as through percutaneous coronary intervention (8–10). However, existing diagnostic methods still have limitations in identifying very early or atypical AMI, and some patients continue to face a high risk of recurrent adverse cardiovascular events even after receiving standard treatment, highlighting the need for further optimization of current diagnostic and therapeutic strategies. Therefore, exploring more sensitive and specific diagnostic biomarkers from the deeper mechanisms of myocardial ischemic injury holds significant scientific and clinical value for early warning, precise diagnosis, and improved prognosis of AMI patients.

Autophagy is a highly conserved intracellular degradation and recycling process that maintains cellular homeostasis by clearing damaged organelles and misfolded proteins (11–13). In the cardiovascular system, moderate autophagy serves as a crucial cytoprotective mechanism for cardiomyocytes in response to stressors such as ischemia and hypoxia. However, excessive or insufficient autophagy can exacerbate myocardial injury and is involved in the pathological processes of heart failure, myocardial hypertrophy, and ischemia-reperfusion injury (14–16). Protein lactylation modification is an emerging, lactate-driven epigenetic modification. Under hypoxic conditions, accumulated intracellular lactate serves as a substrate for enzymes (e.g., lysine acetyltransferases) that catalyze the lactylation of histones or other proteins, thereby directly regulating gene transcription and cellular function (17, 18). Recent studies have shown that this modification is widely involved in processes such as macrophage polarization, tumor metabolism, and embryonic development (19, 20). During myocardial ischemia, the shift in energy metabolism toward anaerobic glycolysis leads to substantial lactate production, which may influence key processes in cardiomyocytes, such as inflammatory responses and apoptosis, through lactylation (21–23). However, the potential association between autophagy and lactylation, as well as their coordinated transcriptional patterns in AMI, remains unclear. Therefore, this study aims to systematically explore the expression signatures of autophagy- and lactylation-related genes (ARGs and LRGs) in AMI through integrated bioinformatics analysis, and to screen for potential diagnostic biomarkers. This research may offer a novel perspective for understanding the molecular pathogenesis of AMI and for discovering new therapeutic targets.

This study uses publicly available transcriptomic data of AMI and integrates established ARGs and LRGs sets to systematically explore the transcriptional associations between autophagy and lactylation in AMI and to identify potential diagnostic biomarkers. Through multi-level bioinformatics approaches, including differential expression analysis, weighted gene co-expression network analysis (WGCNA), protein-protein interaction (PPI) network analysis, machine learning algorithms, and independent cohort expression validation, candidate biomarkers were screened and identified. Subsequently, their diagnostic performance was evaluated, and an in-depth analysis of their potential biological functions and associations with the immune microenvironment was conducted. This study provides a novel analytical perspective for characterizing the relationship between autophagy- and lactylation-related gene signatures in AMI. The identified biomarkers and associated functional insights may offer preliminary clues for future research on AMI and its clinical adjunctive diagnosis.

## 2 Materials and methods

### 2.1 Data acquisition

Publicly available transcriptomic data related to AMI were retrieved from the Gene Expression Omnibus (GEO) database (https://www.ncbi.nlm.nih.gov/geo/) on November 26, 2025. The GSE66360 dataset (platform: GPL570) served as the training cohort for this study, comprising 49 samples of peripheral blood circulating endothelial cells from patients with AMI (AMI group) and 50 samples from gender- and age-matched healthy controls (control group). The GSE48060 dataset (platform: GPL570) was employed as the independent testing cohort, which included 31 AMI and 21 control samples from the same cell type. For raw data preprocessing, log2 transformation was applied to eligible samples based on expression intensity criteria. Probe annotation was performed using the GPL570 platform file. For probes annotated to multiple genes (separated by “///”), only the first gene symbol was retained. Probes without gene annotation or annotated as “—” were excluded. For duplicate genes, the probe with the maximum row mean expression value was retained.

Furthermore, a total of 371 ARGs were retrieved from the Human Autophagy Database (HADb) (https://autophagy.lu/v1/) (Supplementary Table S1). Additionally, 332 lactylation-related genes (LRGs) were compiled from previously published literature (Supplementary Table S2) (24).

### 2.2 WGCNA

To investigate the association of ARGs and LRGs with AMI, significantly differentially expressed ARGs and LRGs (DE-ARGs and DE-LRGs) between the control and AMI groups in the GSE66360 dataset were first screened using the Wilcoxon rank-sum test (*p* < 0.05). Subsequently, the ssGSEA algorithm from the GSVA package (v 1.53.28) (25) was applied to calculate the DE-ARG and DE-LRG activity scores for each sample in GSE66360, resulting in an activity score matrix. The significance of inter-group differences in these activity scores was further assessed using the Wilcoxon rank-sum test (*p* < 0.05). Furthermore, for obtaining the key module genes associated with ARGs and LRGs, the WGCNA package (v 1.73) (26) was utilized to perform WGCNA based on all samples. Specifically, a clustering analysis of all samples was initially performed to check and remove outliers via the hclust function. Thereafter, for establishing a scale-free co-expression gene network, optimal soft threshold was selected according to scale-free R2 (0.85) and mean connectivity (approaching 0). Subsequently, genes were divided into different modules according to the hybrid dynamic tree cutting algorithm criteria (minModuleSize = 200). Afterwards, using the scores of DE-ARGs and DE-LRGs as the traits, Spearman correlation analysis was employed via the stats package (v 4.3.3) (27) to assess associations between module eigengenes (MEs) and the traits [|correlation coefficient (cor)| > 0.3, *p* < 0.05]. The module that showed the strongest positive correlation with both traits was defined as the key module. Next, the module membership (MM) and gene significance (GS) analyses were performed on genes in the key module. Genes with |MM| > 0.8 and |GS| > 0.2 were selected as key module genes.

### 2.3 Differential expression analysis

To obtain genes with differential expression between the control and AMI groups in GSE66360, the limma package (v 3.56.2) (28) was employed for the purpose of discerning DEGs [*p* < 0.05, |log2fold change (FC)| > 0.5]. Furthermore, a volcano plot for DEGs was generated utilizing the ggplot2 package (v 3.5.2) (29), and labeled with the top 10 gene names demonstrating significant up- or down-regulation according to the descending order of |log2FC|, while a heatmap of the labeled DEGs was plotted utilizing the ComplexHeatmap package (v 2.16.0) (30).

### 2.4 Identification and related functional analyses of candidate genes

To further explore genes associated with both autophagy and lactylation in AMI, the ggvenn package (v 0.1.10) (31) was used to detect the intersection of DEGs and key module genes. Genes in the intersection were defined as candidate genes. Subsequently, Gene Ontology (GO) and Kyoto Encyclopedia of Genes and Genomes (KEGG) analyses were performed using the clusterProfiler package (v 4.1.5) (32) to elucidate the biological functions associated with the candidate genes (*p* < 0.05). The enrichment results were sorted in ascending order of the *p*-values, and the top 5 GO terms of each category [biological processes [BPs], molecular functions [MFs], and cellular components [CCs], individually] and the top 10 KEGG terms were respectively displayed.

### 2.5 Determination of biomarkers

To investigate the PPI relationships among the candidate genes, their encoded proteins were first analyzed using the STRING database (https://www.string-db.org) (interaction score > 0.4). Subsequently, the resultant PPI network was visualized and further analyzed with Cytoscape software (v 3.10.1) (33). To identify central nodes within this network, the candidate genes were ranked using 4 distinct algorithms from the CytoHubba plugin: Stress, Betweeness, Edge Percolated Component (EPC), and Closeness. The top 20 genes from each ranking list were extracted, and the intersecting set among these 4 lists was defined as the hub genes. After that, 2 machine learning algorithms were utilized to filter these hub genes in GSE66360. Initially, the randomForest package (v 4.7-1.1) (34) was used to execute the random forest (RF) algorithm with ntree = 74 and mtry = 5 (seed = 10). Genes were sorted by importance, and the top 5 genes were selected as RF characteristic genes. The Boruta algorithm was applied for feature selection using the Boruta package (v 8.0.0) (35), with maxRuns = 500 (seed = 2), to identify Boruta characteristic genes. The intersection of RF and Boruta characteristic genes was generated by the ggvenn package (v 0.1.10), and the overlapping genes were nominated as key genes. Moreover, expression levels of key genes in GSE66360 and GSE48060 were compared between the AMI and control groups utilizing the Wilcoxon rank-sum test (*p* < 0.05). Genes displaying marked differential expression in both sets and exhibiting a consistent trend were defined as biomarkers. To investigate the localization of biomarkers within subcellular compartments, the protein sequences of the biomarkers were retrieved from the National Center for Biotechnology Information (NCBI) database (https://www.ncbi.nlm.nih.gov/) and then submitted to the DeepLoc 2.0 web server (https://services.healthtech.dtu.dk/services/DeepLoc-2.0/) for prediction.

### 2.6 Assessment of biomarker diagnostic performance

To evaluate the potential diagnostic value of the biomarkers for AMI, an artificial neural network (ANN) classification model based on biomarker expression levels was constructed using the GSE66360 dataset as the training set and GSE48060 as the independent validation set. The neuralnet function from the neuralnet package (v 1.44.2) (36) was employed with the group as the dependent variable and biomarkers as independent variables. The model was configured with a single hidden layer containing 10 neurons, a threshold of 0.1, cross-entropy as the loss function, and linear.output = FALSE, with random seeds fixed at 249 for the training set and 17 for the validation set. Receiver operating characteristic (ROC) curves were plotted using the pROC package (v 1.18.5) (37) to calculate the area under the curve (AUC), with an AUC > 0.7 considered indicative of discriminative ability. To further assess the potential clinical application value of the biomarkers in AMI diagnosis, a least absolute shrinkage and selection operator (LASSO) model was constructed using the glmnet package (v 4.1.4) (38) with a fixed random seed of 123. The model was trained via 5-fold cross-validation, with the regularization parameter alpha searched from 0 to 1 at 0.1 intervals. The optimal lambda was selected as lambda.min corresponding to the minimum cross-validation error. The classification decision threshold was optimized by searching from 0.3 to 0.7 at 0.05 intervals. Model evaluation and parameter tuning were performed using the caret package (v 3.1-2) (39). ROC curves and AUC values were generated using the pROC package (v 1.18.5).

### 2.7 Gene set enrichment analysis (GSEA)

To further explore the biological functions of the biomarkers, GSEA was performed. The gene set “c2.cp.v2024.1.Hs.symbols.gmt” was extracted from the Molecular Signatures Database (MSigDB) (https://www.gsea-msigdb.org/gsea/msigdb) as a reference gene set. In the AMI and control groups from GSE66360, the Spearman correlation coefficients of each biomarker with all other genes were calculated via the psych package (v 2.4.6.26) (40). All genes were then ranked in descending order in light of their correlation coefficients. This ranked set of genes was subjected to GSEA using the clusterProfiler package (v 4.1.5) [p.adjust < 0.05, |normalized enrichment score (NES)| > 1]. The enrichment results were sorted in ascending order of p.adjust-values, and the top 5 pathways were selected and visualized.

### 2.8 Immune microenvironment analysis

To characterize the role of specific immune cell subsets in the progression of AMI, in the GSE66360 dataset, the infiltration abundance of 28 immune cell types (41) was analyzed using the ssGSEA algorithm in the GSVA package (v 1.53.28), and a heatmap was plotted to display the results. Subsequently, the Wilcoxon rank-sum test was utilized to compare the differences in immune cell infiltration abundance between the AMI and control groups (*p* < 0.05). Immune cells with significantly different infiltration abundances were screened and designated as differentially infiltrated immune cells. To examine the correlations among differentially infiltrated immune cells and between the biomarkers and these immune cells, Spearman correlation analyses were performed using the stats package (v 4.3.3) (27) [*p* < 0.05, |correlation coefficient (cor)| > 0.3].

### 2.9 Regulatory factor and drug prediction

To construct the upstream regulatory network of the identified biomarkers, a multi-step bioinformatic analysis was performed. Firstly, microRNAs (miRNAs) potentially targeting these biomarkers were retrieved using the multiMiR package (v 1.22.0) (42), which integrates predictions from 14 databases (like miRTarBase, TargetScan, and miRDB). To ensure high confidence, only key miRNAs predicted by at least 4 databases were retained. Subsequently, the ENCORI database (https://rnasysu.com/encori/) was employed to identify long non-coding RNAs (lncRNAs) that could interact with these key miRNAs. A regulatory network encompassing biomarkers, miRNAs, and lncRNAs was then visualized using the igraph package (v 2.0.3) (43). Additionally, therapeutic drugs targeting the biomarkers were identified using the Drug-Gene Interaction Database (DGIdb) (https://dgidb.org/). Only approved drugs with an interaction score greater than 1 were retained for further analysis. These filtered drug-gene interactions were subsequently visualized using Cytoscape software (v 3.10.3).

### 2.10 Induction of AMI rat model via coronary artery ligation

The rat was anesthetized and fixed in a supine position, followed by disinfection of the chest and neck area after hair removal. Endotracheal intubation was performed and connected to a ventilator. A left thoracotomy was carried out through the fourth or fifth intercostal space to expose the heart, and the left anterior descending coronary artery was ligated proximally to induce myocardial infarction. The chest was then closed layer by layer. This study was approved by Shanghai University of Medicine & Health Sciences (Approval No. 2025-NSFC-16-14). Review Date: 18 February 2025.

### 2.11 Reverse transcription quantitative PCR (RT-qPCR) analysis

To verify biomarker expression in the rat models, RT-qPCR analysis was performed. Tissue samples were harvested from 4 control and 4 AMI rats for analysis. Total RNA was extracted from the 8 tissue samples using the TRIzol reagent (Vazyme, China) following the manufacturer's instructions. Subsequently, the RNA concentration was measured with the NanoPhotometer N50. The cDNA was synthesized by reverse transcription using the HP All-in-one qRT Master Mix II RT203-Ver.1 (YoungGen, China), and the reverse transcription was performed with the S1000TM Thermal Cycler (Bio-Rad, USA). The sequences of all primers can be found in Table 1. The qPCR assay was conducted using the CFX Connect Real-time Quantitative Fluorescence PCR Instrument (Bio-Rad, USA). The relative quantification of mRNAs was determined by the 2-*ΔΔ*Ct method. The RT-qPCR results were then analyzed and visualized in GraphPad Prism 10 (https://www.graphpad.com/).

**Table 1:** Primer sequences for RT-qPCR.

Primer	Sequence (5'-3’)
ATM F	CTTAAGGGTTCTCGTCGACCT
ATM R	CAGAACGTGCATCCTCACCT
STAT1 F	CCACAGTTGGACGTTCTGGA
STAT1 R	GTGTCAGTTTGGGGAGTCCTT
GAPDH F	GGCCGGAGACGAATGGAAATTA
GAPDH R	CCAAATCCGTTCACACCGAC

### 2.12 Statistical analysis

Statistical analysis was performed using R software (v 4.2.2). Group comparisons were assessed using the Wilcoxon rank-sum test, with *p*-values adjusted via the Benjamini-Hochberg method (adjusted *p* < 0.05 considered significant). Spearman's rank correlation coefficients were used for correlation analyses. In machine learning procedures, random seeds were fixed and key parameters were detailed in the respective method sections. Model performance was evaluated using ROC curves with AUC, and the LASSO model was trained via 5-fold cross-validation. All tests were two-sided. Detailed parameter settings and analysis code are provided to ensure reproducibility. Significance levels: ****p < 0.0001, ***p < 0.001, **p < 0.01, *p < 0.05.

## 3 Results

### 3.1 Characterization of the MEturquoise and its constituent genes

A total of 122 DE-ARGs and 114 DE-LRGs were identified from GSE66360 (Supplementary Table S3). Subsequent analysis revealed significantly higher activity of DE-ARGs in the AMI group and higher activity of DE-LRGs in the control group (Figure 1A), suggesting a potential involvement of autophagy and lactylation in AMI. Using DE-ARG and DE-LRG activity scores as traits, WGCNA was performed. Cluster analysis confirmed the absence of outlier samples (Figure 1B), and a soft-thresholding power of 16 was selected to achieve approximate scale-free topology (Figure 1C). A co-expression network was then constructed, clustering genes into 4 distinct modules (excluding the grey module) (Figure 1D). Correlation analysis indicated that the MEturquoise showed the strongest positive correlation with both DE-ARG activity scores (co*r* = 0.81, *p* = 3.9e-24) and DE-LRG activity scores (co*r* = 0.91, *p* = 6.5e-39) (Figure 1E). Therefore, the MEturquoise was defined as the key module. Subsequently, 1,442 key module genes were identified from this module based on MM and GS criteria (|MM| > 0.8, |GS| > 0.2) (Figure 1F). These genes likely represent core candidates associated with autophagy and lactylation processes in the context of AMI.

**Figure 1:**
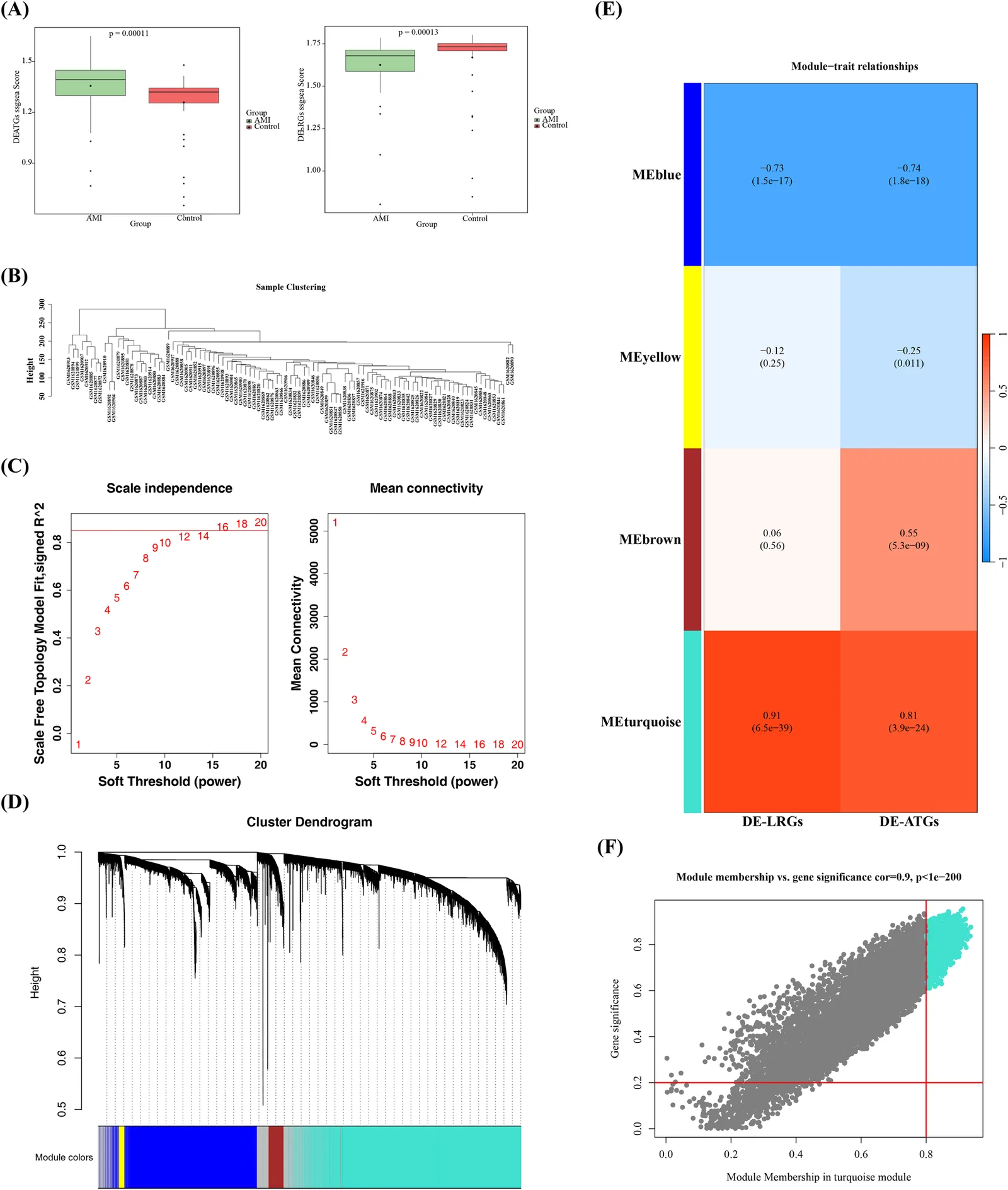
Derivation of 1,442 key module genes from the MEturquoise. **(A)** Box plots showed the activity scores of differentially expressed autophagy-related genes (DE-ARGs) and differentially expressed lactylation-related genes (DE-LRGs) between the control and acute myocardial infarction (AMI) groups; **(B)** Cluster analysis of samples was conducted to assess outlier samples; **(C)** The optimal soft threshold was determined to achieve approximate scale-free topology; **(D)** The gene co-expression network was constructed and visualized; **(E)** The correlations between the 4 modules and the traits (DE-ARG and DE-LRG activity scores) were analyzed; **(F)** Module membership (MM)-gene significance (GS) analysis was performed to identify 1,442 key module genes.

### 3.2 Discovery of 185 candidate genes and investigation into their biological roles

Differential expression analysis identified 2,626 DEGs, comprising 984 up-regulated and 1,642 down-regulated genes in the AMI group compared to controls (Figures 2A,B). Taking the intersection of 2,626 DEGs and 1,442 key module genes yielded 185 candidate genes (Figure 2C). Functional enrichment analysis revealed that these candidate genes were significantly associated with 431 GO terms (368 BPs, 25 CCs, 38 MFs) (Supplementary Table S4). The top 5 of these BPs included “antigen receptor-mediated signaling pathway” and “negative regulation of leukocyte apoptotic process”. The top 5 enriched CCs included “mitochondrial small ribosomal subunit” and “organellar ribosome”. Meanwhile, the top 5 of these MFs included “phosphatase binding” and “ubiquitin-like protein ligase binding” (Figure 2D). In addition, KEGG enrichment analysis showed 185 candidate genes were collectively enriched in 45 pathways (*p* < 0.05) (Supplementary Table S5), and the top 10 pathways included “Th17 cell differentiation” and “NF-kappa B signaling pathway” (Figure 2E). Through GO and KEGG analyses, these candidate genes were shown to correlate with multiple critical signaling pathways.

**Figure 2:**
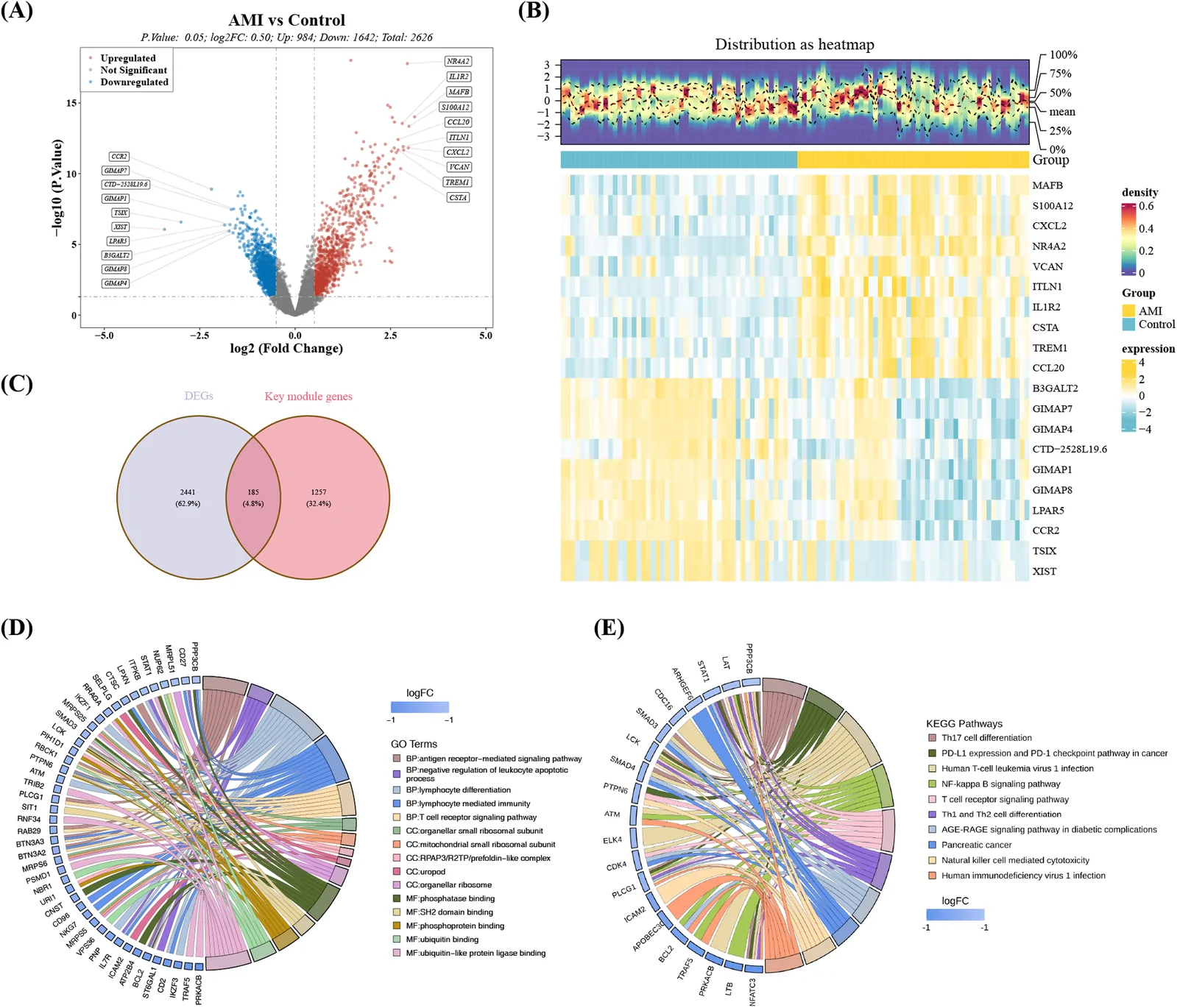
Discovery of 185 candidate genes and investigation into their biological roles. **(A,B)** A volcano plot and a heatmap of differentially expressed genes (DEGs) between the AMI and control groups in GSE66360; **(C)** A Venn diagram was used to identify 185 candidate genes by intersecting DEGs with key module genes; **(D,E)** Gene Ontology (GO) and Kyoto Encyclopedia of Genes and Genomes (KEGG) enrichment analyses were performed to explore the biological roles of the 185 candidate genes.

### 3.3 Acquisition of 2 biomarkers: ATM and STAT1

As shown in Figure 3A, the PPI network revealed interactions among proteins encoded by 126 of the 185 candidate genes, while NCOA5 and VPS36 exhibited extremely high connectivity and might play an important regulatory role in the network. Subsequently, 20 genes were screened out by each of the Stress, Betweenness, EPC, and Closeness algorithms respectively (Figure 3B). After taking the intersection of the screening results from the 4 algorithms, 11 hub genes were obtained (Figure 3C), namely LCK, CD2, STAT1, BCL2, IL7R, IKZF1, FLT3LG, TRAF3IP3, PLCG1, CDK4, ATM. To pinpoint the most predictive genes, machine learning was applied. In the RF model, the top 5 genes ranked by importance were PLCG1, IL7R, ATM, TRAF3IP3, and STAT1 (Figure 3D). The Boruta algorithm identified TRAF3IP3, PLCG1, IL7R, BCL2, ATM, CD2, FLT3LG, STAT1, and LCK as confirmed features (Figure 3E). The intersection of both methods yielded 5 key genes: IL7R, STAT1, TRAF3IP3, PLCG1, and ATM (Figure 3F). Among these, only ATM and STAT1 showed consistently higher expression in the control groups compared to the AMI groups across both the GSE66360 and GSE48060 datasets (Figure 3G). Consequently, ATM and STAT1 were identified as biomarkers associated with both autophagy and lactylation activity scores in AMI. Subcellular localization analysis revealed that ATM was primarily localized in the nucleus, whereas STAT1 was present in both the cytoplasm and the nucleus (Figure 3H). These findings suggest that ATM and STAT1 may serve as potential molecular links between autophagic processes and lactate metabolism/lactylation-related signaling, although direct evidence of their lactylation status during AMI requires further experimental validation.

**Figure 3:**
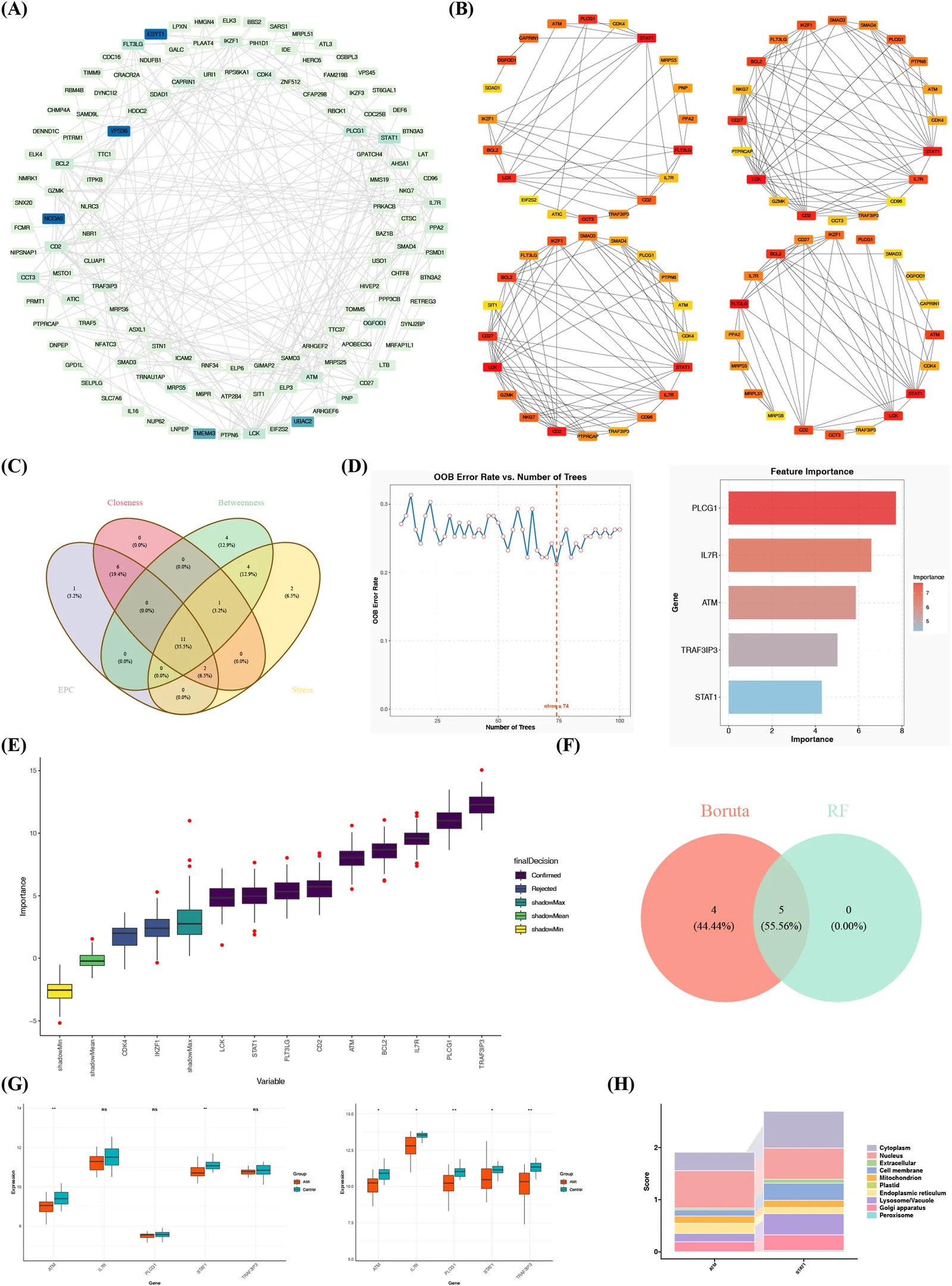
Acquisition of 2 biomarkers: ATM and STAT1. **(A)** The protein-protein interaction (PPI) network of candidate genes was constructed and visualized; **(B)** Results of four distinct algorithms [Stress, Betweeness, Edge Percolated Component (EPC), and Closeness] from the CytoHubba plugin were presented, which were used to screen hub genes; **(C)** The intersection of the results from the 4 distinct algorithms was analyzed to identify hub genes; **(D)** Plots showed the error rate curve and feature importance ranking of genes from the random forest (RF) model; **(E)** The results of the Boruta algorithm were displayed to confirm feature genes; **(F)** A Venn diagram was used to identify key genes by intersecting the results from the two algorithms (RF model and Boruta algorithm); **(G)** Box plots showed the expression levels of key genes in the GSE66360 and GSE48060 datasets; **(H)** Analysis of the subcellular localization of ATM and STAT1 was performed and presented.

### 3.4 Confirmation of ATM and STAT1 as effective diagnostic biomarkers

The ANN model constructed based on ATM and STAT1 demonstrated a strong ability to distinguish AMI from control samples in both the GSE66360 (AUC = 0.934) and GSE48060 (AUC = 0.892) datasets (Figures 4A,B). Similarly, the confusion matrices indicated that the LASSO model also constructed with ATM and STAT1 achieved effective discrimination and potential clinical utility. In GSE66360 (AUC = 0.760), the model showed a true negative rate of 31.3%, a true positive rate of 41.4%, and an overall accuracy of 0.727 (Figures 4C,D). Corresponding results in GSE48060 (AUC = 0.771) included a true negative rate of 32.7%, a true positive rate of 40.4%, and an accuracy of 0.731 (Figures 4E,F). Together, these results consistently support the combination of ATM and STAT1 as effective biomarkers for AMI diagnosis, highlighting their reliable performance across independent cohorts and different modeling approaches.

**Figure 4:**
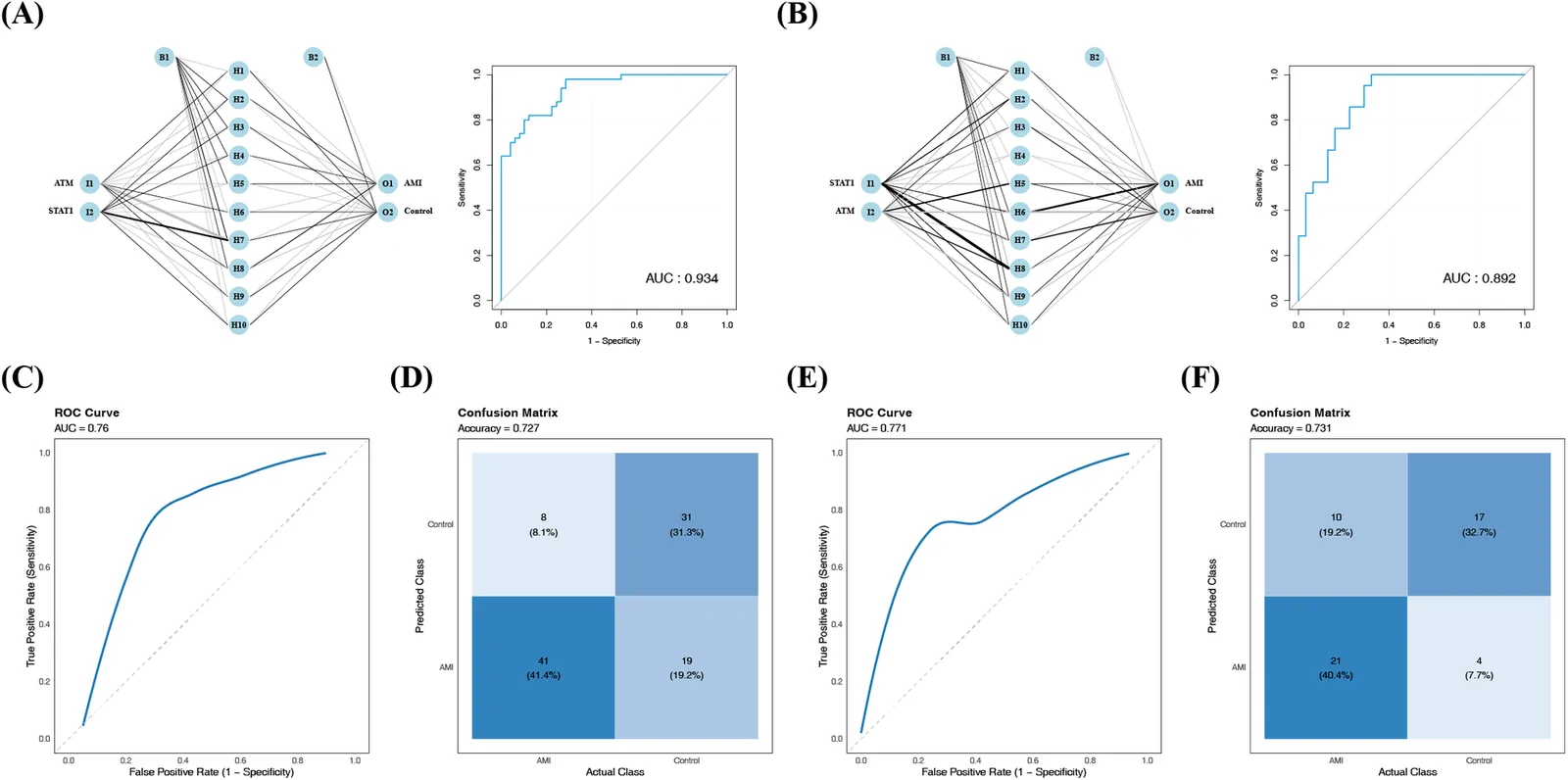
Confirmation of ATM and STAT1 as effective diagnostic biomarkers. **(A,B)** Artificial neural network (ANN) models based on ATM and STAT1, each containing one input layer, one hidden layer, and one output layer, were constructed for AMI diagnosis; receiver operating characteristic (ROC) curves of the ANN models in GSE66360 and GSE48060 were plotted to evaluate diagnostic performance. **(C–F)** ROC analyses of the least absolute shrinkage and selection operator (LASSO) model and confusion matrices were performed in GSE66360 and GSE48060, and the results were presented in these panels.

### 3.5 Functional characterization of ATM and STAT1

Comprehensive functional characterization of the two biomarkers was performed using Gene Set Enrichment Analysis (GSEA). The results revealed that ATM was enriched in 1,644 pathways and STAT1 in 1,615 pathways (Supplementary Table S6). Notably, the top 5 most significantly enriched pathways for both ATM and STAT1 were identical, including “KEGG neuroactive ligand-receptor interaction”, “REACTOME olfactory signaling pathway”, “KEGG olfactory transduction”, “REACTOME translation”, and “REACTOME keratinization” (Figures 5A,B). This substantial overlap in enriched pathways strongly suggested that ATM and STAT1 may function within closely related biological processes, potentially contributing to shared mechanisms underlying AMI pathogenesis.

**Figure 5:**
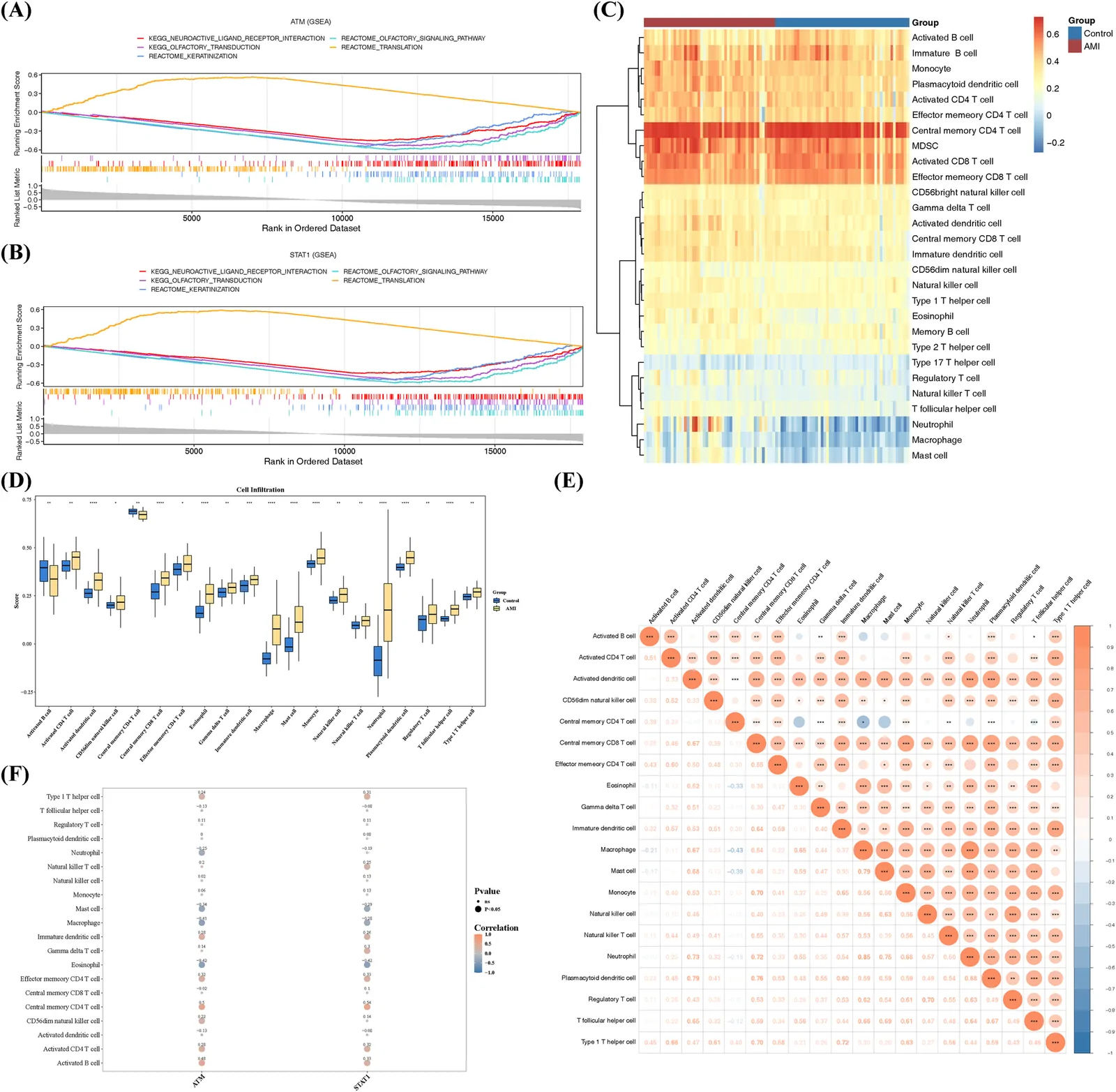
Functional enrichment and immune microenvironment analyses. **(A,B)** Gene set enrichment analysis (GSEA) of ATM **(A)** and STAT1 **(B)** was performed to explore their enriched pathways, and the results were presented in these panels; **(C)** A heatmap of the infiltration levels of 28 immune cell types in GSE66360 was plotted to show their distribution patterns; **(D)** Box plots of the infiltration levels of 20 differentially infiltrated immune cells between the control and AMI groups were plotted to compare their group differences; **(E)** Correlation analyses of the differentially infiltrated immune cells were conducted to evaluate their interrelationships; **(F)** Correlation analyses between the biomarkers (ATM and STAT1) and the differentially infiltrated immune cells were performed.

### 3.6 Analysis of the immune microenvironment in AMI

To characterize the immune microenvironment in AMI, immune cell infiltration was further analyzed. The infiltration levels of 28 immune cell types were assessed between the AMI and control groups, with central memory CD4T cells showing high infiltration abundance in both groups (Figure 5C). Then, 20 immune cell types were identified with significantly different infiltration levels between AMI and control samples. Interestingly, only activated B cells and central memory CD4T cells exhibited higher infiltration in the control group, while the other 18 cell types, including central memory CD8T cells, were more abundant in the AMI group (Figure 5D). Correlation analysis revealed the strongest positive correlation between neutrophils and macrophages (co*r* = 0.85, *p* < 0.001), whereas a significant negative correlation was observed between macrophages and central memory CD4T cells (cor = −0.43, *p* < 0.05) (Figure 5E). Furthermore, ATM showed a significant positive correlation with activated B cells (co*r* = 0.48, *p* < 0.05) and a negative correlation with eosinophils (cor = −0.42, *p* < 0.05). STAT1 was positively correlated with central memory CD4T cells (co*r* = 0.54, *p* < 0.05) and negatively correlated with eosinophils (cor = −0.42, *p* < 0.05) (Figure 5F). These correlation patterns suggested a potential association between the immune landscape and the autophagy- and lactylation-related gene expression signatures represented by ATM and STAT1 in AMI pathogenesis.

### 3.7 Regulatory network analysis and therapeutic drug prediction for biomarkers

Subsequent analysis explored the regulatory networks of the biomarkers. Predictions identified 18 miRNAs (e.g., miR-18a-5p and miR-421) and 33 lncRNAs (e.g., GAS5 and HCG11) potentially targeting ATM (Figure 6A). For STAT1, 7 miRNAs (e.g., miR-150-5p and miR-27b-3p) and 18 lncRNAs (e.g., PWAR5 and LINC01128) were predicted (Figure 6B). Notably, several lncRNAs, including XIST and NEAT1, were found to potentially regulate both ATM and STAT1. Furthermore, drug prediction analysis indicated that the approved Tuvusertib and ATR inhibitor ART0380 could target ATM, while Picoplatin, Guttiferone K, and Garcinol were predicted to target STAT1 (Figure 6C). These results reveal a multi-layer regulatory landscape and highlight repurposable drug candidates, providing mechanistic and therapeutic insights into the roles of ATM and STAT1 in AMI.

**Figure 6:**
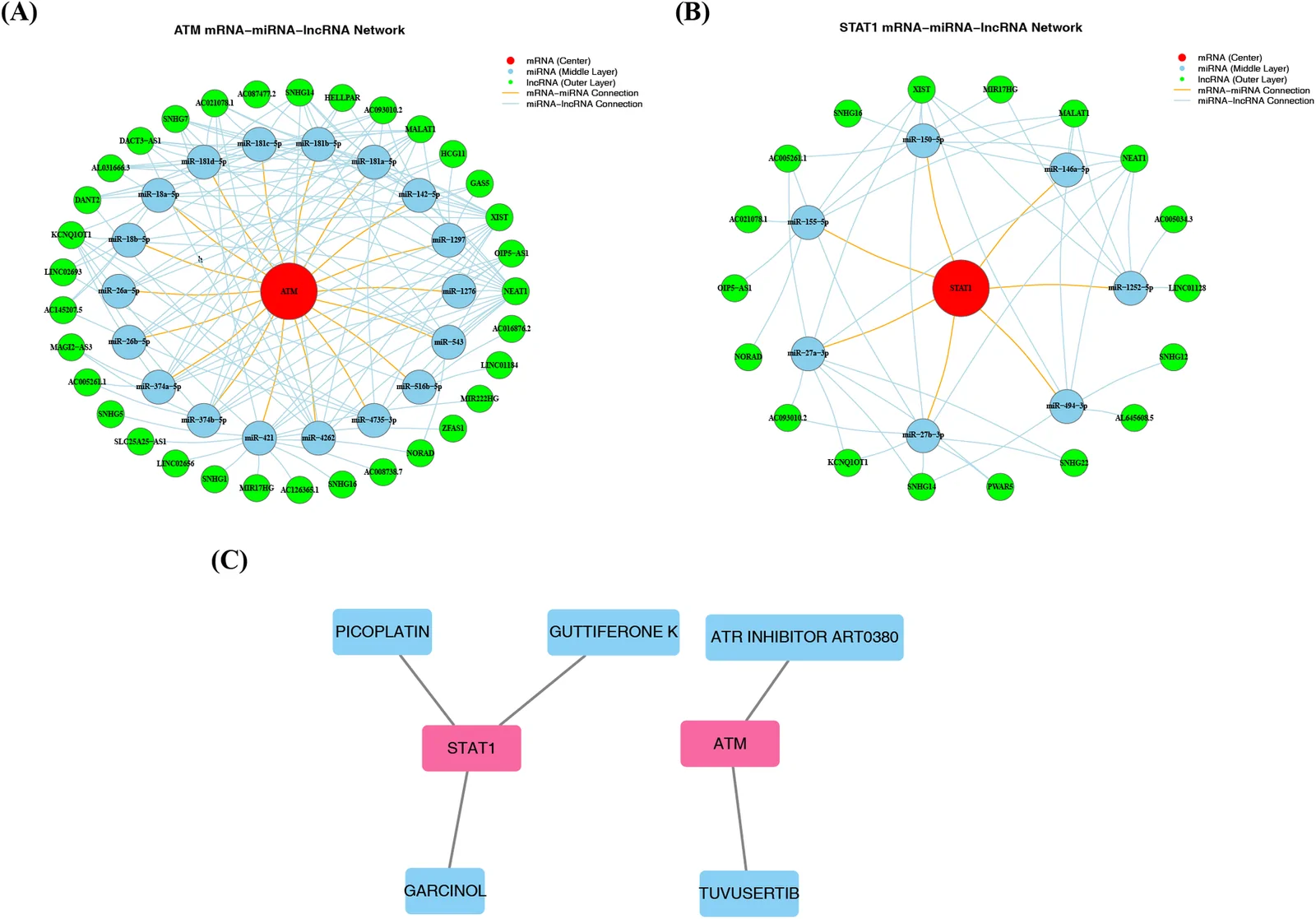
Exploration of the regulatory networks and drug prediction. **(A,B)** The mRNA-microRNA (miRNA)-long non-coding RNA (lncRNA) regulatory networks for ATM **(A)** and STAT1 **(B)** were constructed and visualized to show their upstream regulatory relationships; **(C)** Drug prediction analysis targeting ATM and STAT1 was performed to identify potential therapeutic drugs.

### 3.8 The expression of ATM and STAT1

Subsequently, the expression levels of 2 biomarkers (ATM and STAT1) were investigated in rat models of AMI and control tissue samples. The RT-qPCR results showed that compared with the control group, the expression level of both biomarkers were remarkably downregulated in the AMI group (Figures 7A,B), which was consistent with the data from the GSE70362 and GSE176205 datasets. These findings, together with the bioinformatics screening, suggest ATM and STAT1 as potential biomarkers for AMI, with their downregulation in AMI observed at the transcriptional level in a limited rat model (*n* = 4 per group); however, these preliminary results require confirmation in larger animal cohorts.

**Figure 7:**
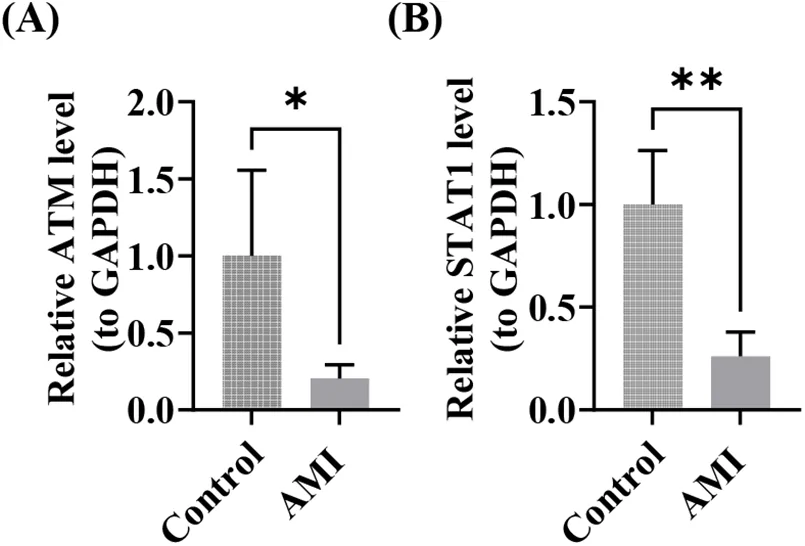
The reverse transcription quantitative PCR (RT-qPCR) analysis. **(A,B)** The expression of ATM **(A)** and STAT1 **(B)** in rat model tissue samples.

## 4 Discussion

Cardiomyocytes face severe ischemia, hypoxia, and metabolic crisis following AMI. In this pathological process, dysfunctional autophagy has been confirmed to be closely associated with cardiomyocyte death and adverse remodeling (15, 44). Concurrently, heightened anaerobic glycolysis induced by hypoxia leads to substantial lactate accumulation, which influences key processes in cardiomyocytes, such as inflammatory responses and apoptosis (22, 45). However, the crosstalk and coordinated regulatory network between autophagic activity and lactylation-related gene expression signatures in AMI remain largely unexplored. Based on this, the present study integrated publicly available transcriptomic data of AMI with known gene sets related to autophagy and lactylation. Through systematic bioinformatic analysis, ATM and STAT1 were identified as key nodal molecules potentially bridging autophagic processes and lactylation-associated transcriptional reprogramming in AMI. Evaluation results demonstrated that a diagnostic model constructed based on these two genes exhibited good discriminatory performance in two independent cohorts, suggesting their potential as novel composite biomarkers. Further mechanistic exploration revealed that ATM and STAT1 are co-enriched in signaling pathways such as “neuroactive ligand-receptor interaction,” and both show a consistent and significant negative correlation with the infiltration level of eosinophils in the immune microenvironment. These findings indicate that ATM and STAT1 may not only reflect metabolic-autophagic stress at the transcriptomic level under AMI but also correlate with alterations in the local immune microenvironment, particularly the infiltration of specific immune cells. Thus, they may serve as potential molecular bridges between autophagy- and lactylation-related gene expression signatures and systemic inflammatory responses, offering new perspectives and potential intervention targets for understanding the complex pathophysiological network of AMI.

Ataxia telangiectasia mutated (ATM) is a serine/threonine kinase whose known functions include the regulation of DNA-damage repair, autophagy, oxidative stress, and inflammatory signal transduction (46–50). In the cardiovascular system, ATM participates in the repair of myocardial ischemic injury and the maintenance of vascular homeostasis. For instance, in failing hearts, activation of the ATM-mediated DNA-damage response pathway promotes the degradation of LARP7, resulting in impaired mitochondrial biogenesis and elevated oxidative stress. Conversely, treatment with small-molecule ATM inhibitors after acute myocardial infarction (AMI) restores LARP7 expression and improves cardiac function (51). Consistent with the above-mentioned protective effects, the present study observed significant down-regulation of ATM expression in patients with AMI. This finding is further supported by evidence that ATM mutations can lead to endothelial dysfunction, enhanced thrombotic tendency, and increased susceptibility to atherosclerosis (50), whereas ATM deficiency exacerbates cardiac dysfunction following AMI (52, 53). Collectively, these lines of evidence suggest that down-regulated ATM expression in AMI may drive disease progression by impairing DNA-damage repair, disrupting mitochondrial homeostasis, and dysregulating immune responses. Nevertheless, since the results of the present study are derived from transcriptomic analyses of circulating endothelial cells, the functional consequences of ATM down-regulation in cardiomyocytes and other cardiac cell types remain to be experimentally validated.

Signal transducer and activator of transcription 1 (STAT1), a transcription factor of the STAT family, is known to mediate signal transduction of cytokines such as interferons (IFNs) and interleukins, and regulate the expression of genes involved in immune-inflammatory responses, cell proliferation and differentiation (54–56). In the cardiovascular system, STAT1 acts primarily as an inflammatory regulator, and its dysfunction is closely associated with the progression of atherosclerosis and myocardial fibrosis (57–62). The present study found that STAT1 expression was significantly down-regulated in patients with acute myocardial infarction (AMI). Previous studies have demonstrated that STAT1 activation promotes the expression of inflammatory factors including TNF-*α* and CXCL10, recruits infiltration of macrophages and T lymphocytes, and accelerates lipid deposition and fibrous cap degradation (57–59). In addition, STAT1 can up-regulate the expression of fibrosis-related genes and facilitate the proliferation of myocardial fibroblasts as well as collagen deposition (60–62). Nevertheless, these pro-inflammatory and pro-fibrotic effects were mainly observed under conditions of STAT1 activation. In our cohort, STAT1 expression was down-regulated, which may reflect a transcriptional state distinct from the classical activation pattern in AMI, rather than a simple loss-of-function. Moreover, STAT1 drives disease progression by inhibiting autophagy in cardiac hypertrophy models (62), whereas autophagy has been proven to exert protective effects in AMI (15, 44). Accordingly, down-regulation of STAT1 in AMI may be accompanied by a relative increase in autophagic activity, though its functional consequences require further analysis in the context of specific cell types and pathological stages. It should be noted that the above speculations are derived solely from transcriptomic correlation analyses, and the differential effects of STAT1 down-regulation on cardiomyocytes, endothelial cells and immune cells remain to be directly verified by in-vitro and in-vivo experiments.

However, the potential interplay between ATM/STAT1 and lactylation-related transcriptional programs remains unclear. This study found that both ATM and STAT1 belong to a key module highly correlated with the activity scores of lactylation-related gene sets. This provides a novel associative perspective for understanding the crosstalk between metabolic remodeling and cellular stress/immune signaling in AMI at the transcriptomic level. It suggests that future research could focus on elucidating the molecular basis of this association, particularly whether lactate metabolism influences ATM/STAT1 expression or downstream transcriptional targets, which may represent a previously unrecognized link between the metabolic microenvironment and cellular responses in AMI.

Immune infiltration and correlation analysis provide a more refined immunological perspective. The positive correlation between ATM expression levels and activated B cell infiltration suggests that, post-AMI, a moderate B-cell response may help clear necrotic debris and generate protective antibodies (63, 64). Central memory CD4+ T cells, positively correlated with STAT1, are associated with faster and more coordinated immune memory responses (65, 66). Their reduced infiltration levels observed in AMI patients may reflect a weakened beneficial immune regulatory capacity. Both ATM and STAT1 show significant negative correlations with eosinophils. Recent studies have found that eosinophil infiltration is associated with atherosclerotic plaque vulnerability and worsened myocardial fibrosis (67, 68). However, as these correlations do not establish causal relationships, the possibility that ATM/STAT1 downregulation is associated with eosinophil infiltration, rather than directly suppressing it, should be considered. Nonetheless, the observed negative correlation highlights a potential link between ATM/STAT1 expression and eosinophil-related inflammatory processes that warrants further investigation.

In summary, this study implicates ATM and STAT1 as key molecules associated with autophagy and lactylation-related transcriptional programs in the AMI pathological network, highlighting their potential involvement in metabolic remodeling, cellular stress responses, and immune inflammation. Future studies should utilize experimental approaches to investigate whether and how lactate metabolism influences the expression or functional activity of these two genes in both *in vivo* and *in vitro* models, and to clarify their specific roles in modulating the infiltration of specific immune cell subsets. This would provide new targets for the precise diagnosis and treatment of AMI, extending beyond traditional hemodynamic interventions.

## 5 Conclusions

This integrated bioinformatics analysis has, for the first time, systematically revealed the potential crosstalk between autophagy- and lactylation-related gene expression signatures in AMI, identifying ATM and STAT1 as key molecules associated with this network. The diagnostic model based on these two genes demonstrates promising potential. Moreover, functional enrichment and immune microenvironment correlation analyses offer novel insights into the molecular pathogenesis of AMI. However, this study has several limitations. First, the conclusions are primarily derived from retrospective bioinformatics analysis of public databases. Although validated in independent cohorts, the clinical utility of ATM/STAT1 as biomarkers and their association with lactylation-related transcriptional programs require further confirmation in prospective clinical cohorts and experimental models. Second, the animal validation included only 4 rats per group, a small sample size that may affect statistical power and reproducibility; thus, the observed downregulation of ATM and STAT1 in AMI warrants validation in larger animal studies and clinical cohorts. Third, the observed gene co-expression and correlations do not establish definitive causal relationships. For example, the mechanisms by which ATM and STAT1 influence immune cell infiltration (e.g., eosinophils) and whether lactate metabolism or lactylation-associated signals regulate their expression or function remain to be elucidated. Future work will employ *in vitro* (e.g., cardiomyocyte hypoxia) and *in vivo* models to examine how ATM/STAT1 silencing or overexpression affects autophagy, lactate metabolism, and cardiomyocyte survival. Additionally, we plan to profile the global lactylation landscape in AMI to identify potential lactylated proteins and to better understand the interplay between metabolic stress and cellular signaling. Ultimately, we aim to clarify the roles of ATM and STAT1 in bridging metabolic stress and immune inflammation, thereby providing a stronger experimental foundation for AMI mechanistic research and precision therapy.

## Data Availability

The datasets presented in this study can be found in online repositories. The names of the repository/repositories and accession number(s) can be found in the article/Supplementary Material.

## Supplementary material

Supplementary Table 1 Summary of ARGs.

Supplementary Table 2 Summary of LRGs.

Supplementary Table 3 Summary of DE-ARGs and DE-LRGs.

Supplementary Table 4 Summary of GO analysis.

Supplementary Table 5 Summary of KEGG analysis.

Supplementary Table 6 Summary Table of GSEA for ATM and STAT1.

Supplementary Figure 1 Pearson correlation between the activity scores of ARGs/LRGs (used as traits) and distinct gene modules in the GSE66360 dataset. The vertical axis represents different gene modules, and the horizontal axis indicates the corresponding traits. Values above represent correlation coefficients, and the values in parentheses denote *p* values.
