# Supplementary material for: Integrating transcriptomic analysis and experimental validation unravels autophagy- and lactylation-related gene signatures in acute myocardial infarction
Source: Front Cardiovasc Med. 2026 Oct 7;13:1814910. doi: 10.3389/fcvm.2026.1814910 (PMC13683661; doi:10.3389/fcvm.2026.1814910)
Supplement: Supplementary file 1 [file Table7.docx]

Supplementary Material

**1 Supplementary Tables**

**Supplementary Table 1: Summary of ARGs;**

**Supplementary Table 2: Summary of LRGs;**

**Supplementary Table 3: Summary of DE-ARGs and DE-LRGs;**

**Supplementary Table 4: Summary of GO analysis;**

**Supplementary Table 5: Summary of KEGG analysis;**

**Supplementary Table 6: Summary Table of GSEA for ATM and STAT1.**

**2 Supplementary Figures**

**Supplementary Figure 1** Pearson correlation between the activity scores of ARGs/LRGs (used as traits) and distinct gene modules in the GSE66360 dataset. The vertical axis represents different gene modules, and the horizontal axis indicates the corresponding traits. Values above represent correlation coefficients, and the values in parentheses denote p values.
